# Intrinsic capacity trajectories and socioeconomic inequalities in health: the contributions of wealth, education, gender, and ethnicity

**DOI:** 10.1186/s12939-024-02136-0

**Published:** 2024-03-11

**Authors:** Aaron Salinas-Rodríguez, Julián Alfredo Fernández-Niño, Ana Rivera-Almaraz, Betty Manrique-Espinoza

**Affiliations:** 1 Center for Evaluation and Surveys Research, National Institute of Public Health, Cuernavaca, Morelos Mexico; 2 Department of International Health, Johns Hopkins Bloomberg School of Public Health, 615 N. Wolfe Street, Room E8532, Baltimore, MD 21205 USA; 3 Department of Public Health, Universidad del Norte, Barranquilla, Atlántico Colombia

**Keywords:** Intrinsic capacity, Healthy aging, Health inequalities, Social determinants of health

## Abstract

**Background:**

Life-long health inequalities exert enduring impacts and are governed by social determinants crucial for achieving healthy aging. A fundamental aspect of healthy aging, intrinsic capacity, is the primary focus of this study. Our objective is to evaluate the social inequalities connected with the trajectories of intrinsic capacity, shedding light on the impacts of socioeconomic position, gender, and ethnicity.

**Methods:**

Our dynamic cohort study was rooted in three waves (2009, 2014, 2017) of the World Health Organization’s Study on Global AGEing and Adult Health in Mexico. We incorporated a nationally representative sample comprising 2722 older Mexican adults aged 50 years and over. Baseline measurements of socioeconomic position, gender, and ethnicity acted as the exposure variables. We evaluated intrinsic capacity across five domains: cognition, psychological, sensory, vitality, and locomotion. The Relative Index of Inequality and Slope Index of Inequality were used to quantify socioeconomic disparities.

**Results:**

We discerned three distinct intrinsic capacity trajectories: steep decline, moderate decline, and slight increase. Significant disparities based on wealth, educational level, gender, and ethnicity were observed. Older adults with higher wealth and education typically exhibited a trajectory of moderate decrease or slight increase in intrinsic capacity. In stark contrast, women and indigenous individuals were more likely to experience a steeply declining trajectory.

**Conclusions:**

These findings underscore the pressing need to address social determinants, minimize gender and ethnic discrimination to ensure equal access to resources and opportunities across the lifespan. It is imperative for policies and interventions to prioritize these social determinants in order to promote healthy aging and alleviate health disparities. This approach will ensure that specific demographic groups receive customized support to sustain their intrinsic capacity during their elder years.

**Supplementary Information:**

The online version contains supplementary material available at 10.1186/s12939-024-02136-0.

## Background

Inequalities in health are expressed throughout the life course, and it is known that the social determinants that affect health in early stages can also affect it in later stages of life so that they could impact the achievement of healthy aging [1, 2]. According to the World Health Organization (WHO), healthy aging refers to developing and maintaining functional capacity that allows older adults to have well-being in old age. It includes all the multidimensional attributes of human health, including the ability to be and do what they consider most relevant to their lives [3]. In this definition, the emphasis is no longer on the absence of disease or disability but on the ability to maintain independence to carry out their daily activities and highlights the synergies between the individual and its environment. It also stands out the importance of considering that the specific environment in which individuals live depends on society’s social and economic resources during the entire life course. Thus, the functional capacity is determined by individual and environmental characteristics from the early stages of life [4].

The 2015 WHO World Report on Ageing and Health also introduced the concept of intrinsic capacity (IC) to refer to the composite of physical and mental abilities that individuals may draw upon as they age [5]. Within the framework of healthy aging, IC represents individual characteristics determined by genetic inheritance, physiological changes associated with aging, health status, and health-related behaviors. The IC is a holistic concept and positive health measure (rather than an indicator of disease or deficiencies) that makes it possible to measure and monitor health comprehensively and thus better guide healthy aging policies and programs, including preventive strategies at the individual level and public health policies [6].

It is well-established that aging is a dynamic and heterogeneous process. Previous studies have shown that there is no single profile or trend associated with aging, but that healthy aging is expressed in different trajectories because individuals’ socioeconomic and health conditions are not constant but vary during life [7–9]. In contrast, IC trajectories have been less explored, although recent studies have reported a highly heterogeneity among IC trajectories [10–12].

Considering that IC is not constant but is shaped throughout life, specifically by personal and health characteristics, and these attributes cannot be separated from the social context, it is expected that the social determinants of health influence, directly or indirectly, the configuration of IC trajectories. Additionally, the differences (economic, ethnic, or by gender) between social groups associated with the IC and its trajectories could generate health inequities since they are unfair inequalities that would be avoidable through public health interventions and the reduction of structural inequities.

However, evidence on the association between healthy aging trajectories and socioeconomic inequalities in health with longitudinal data is scarce and null for intrinsic capacity. Few previous studies in high-income countries have shown that a better economic position is associated with successful aging [13] or that a lower socioeconomic status is related to the acceleration of aging [14]. In that sense, our study adds to the current body of knowledge in at least two ways. First, no studies have longitudinally explored health inequalities related to intrinsic capacity. Second, studies that have analyzed socioeconomic inequalities in health (cross-sectionally or longitudinally) have focused on economic indicators (income, wealth, education, etc.) and have not explored inequalities associated with gender or ethnicity.

Due to the above, it is essential to identify the potential social determinants of healthy aging trajectories in general and of intrinsic capacity in particular [15]. It is known that social determinants shape socioeconomic inequalities in health, which refer to systematic differences in the health of groups that occupy unequal positions in society [16, 17]. So, determinants like wealth, education, gender, and ethnicity configure inequalities in access to psychosocial, cognitive, economic, and nutritional resources and health services. These differences also influence living conditions and lifestyles and imply greater exposure to environmental risks [18]. All of these elements together affect functional capacity throughout life and create unfair disparities that may inhibit the achievement of healthy aging and the maintenance of IC for some specific groups defined by wealth, ethnicity, or gender. Therefore, this study aimed to analyze the social inequalities associated with IC trajectories in a representative national sample of older Mexican adults. We hypothesize that socioeconomic inequalities in health are still reflected in the most inherent component of healthy aging, namely the intrinsic capacity.

## Methods

### Study design and sample

We used data from three waves of the World Health Organization (WHO) Study on global AGEing and adult health (SAGE) in Mexico. SAGE, a multi-country study, was based on nationally representative samples of individuals aged 50+ years in China, Ghana, India, Mexico, Russia, and South Africa. The study aims and design have been published elsewhere [19]. The SAGE-Mexico study and sample (cross-sectional and longitudinal) have been previously described [20, 21]. Briefly, Wave 1 (baseline data) was collected in 2009 with a sample of 2,404 respondents. Wave 2 was carried out in 2014, with 618 new interviews, and Wave 3, in 2017, with 2,937 participants (including 255 new interviews). 3,277 individuals were interviewed during the three waves. The analytical sample consisted of 2,722 older adults in whom IC trajectories could be estimated because they have at least two longitudinal measurements (Supplemental Fig. 1). Baseline differences between included and excluded participants were observed. The latter were older with a higher prevalence of frailty, disability, and multimorbidity (*p* < 0.05).

#### Outcome

The construction of the metric for measuring IC and estimating its longitudinal trajectories have been described in detail previously [10]. In summary, we applied the Item Response Theory (IRT) to generate a global score for IC (assessing its five domains: cognition, psychological sensory, vitality, and locomotion). The specific variables we used to generate the IC score are described in detail in Supplemental file (Supplemental S1). Three trajectories were identified using the growth mixture models (GMM): low baseline IC with a steep decrease, medium baseline IC with a slightly decreasing, and high baseline IC with a mild increase.

#### Main exposures

We used three domains to characterize health inequities: socioeconomic position (SEP), gender, and ethnicity, measured at baseline.

##### SEP

Educational level and wealth were the indicators for this domain. A household wealth index was derived using the WHO standard approach to estimate permanent income from the ownership of durable goods, dwelling characteristics (type of floors, walls, and cooking stove), and access to water, sanitation, and electricity services [22]. Supplemental Table 1 shows the complete list of durable goods, dwelling characteristics, and services we include in calculating the household wealth index. The index was transformed into quintiles, with the lowest quintile (Q1), indicating the poorest households and the highest quintile (Q5) the richest. We used the years of formal education to estimate the educational level. This variable was also categorized into quintiles (cut points: 0, 3, 6, 9), where Q1 indicates the lowest education level, and Q5 the highest.

##### Gender

A dichotomous variable was formed, with the female being the reference category.

##### Ethnicity

According to the definition of Mexican National Indigenous Institute, the older adults who reported that their mother spoke an indigenous language or defined themselves as belonging to one of the 56 ethnic groups in the country, were considered indigenous, and the rest as non-indigenous.

##### Covariates

The following health and socioeconomic variables were used as potential confounders: age, marital status (with couple = 1), having a paid job, and health insurance (yes = 1). Multimorbidity was included as a dichotomous variable defined as the presence of two or more chronic non-communicable conditions from the list of nine chronic diseases in the SAGE study. The operational definitions of these diseases have been published elsewhere [23]. Physical activity was assessed with the Global Physical Activity Questionnaire (GPAQ), classifying older adults into three categories (low, moderate, and high physical activity) based on reported time spent in moderate or vigorous activities during work, recreational/leisure time, and transportation [24]. Sedentary behavior was measured as a continuous variable considering the daily sitting hours. Tobacco use (never; ever smoked, no longer; current smoker, not daily; current smoker, daily), alcohol consumption (never; ever drinker, no longer; current drinker, low risk; current drinker, high risk), and intake of fruits/vegetables (daily portions: 0–2, 3–4, 5–6 and > 7) were self-reported.

### Statistical analysis

The baseline characteristics of the participants were described using arithmetic means, standard deviations (SD), and proportions where appropriate. We used Chi-square or ANOVA tests to compare the health and sociodemographic characteristics according to the IC trajectories.

We used two indices to estimate the magnitude of socioeconomic inequalities related to IC trajectories (absolute and relative): The relative Index of Inequality (RII) and the Slope Index of Inequality (SII). RII and SII are continuous measures, with higher values indicating greater socioeconomic inequalities in health. We applied generalized linear models with a logarithmic link function to calculate RII and an identity link function to calculate SII. In the first case, the estimated parameters are interpreted as rate ratios and rate differences in the second [25, 26]. For each indicator, the RII and SII were estimated as follows: wealth, quintile 5 (richest) vs. quintile 1 (poorest); educational level, quintile 5 (highest) vs. quintile 1 (lowest); gender, male vs. female; and ethnicity, non-indigenous vs. indigenous. In all cases, comparisons for the IC trajectories were moderate decreasing versus steep decreasing, and slight increasing versus steep decreasing. The specification of these models included the baseline values of the exposure variables (SEP, gender, ethnicity) and the IC trajectories generated from the longitudinal measurements of 3 waves of the SAGE-Mexico study.

We also depicted graphically the inequalities related to IC trajectories for each exposure variable. Firstly, we adjusted a multinomial logistic regression with the IC trajectories as the outcome variable and estimated the conditional probability of being in each trajectory given the groups defined by the categories of wealth, education level, gender, and ethnicity. Secondly, we graphed these results in an *equiplot*, which shows the distance between groups and represents the absolute inequality between them [27].

We further explored interactions between our four exposure variables to identify possible subgroups of the most vulnerable older adults (women and indigenous, lower wealth and lower education, etc.). We assessed the statistical significance of these potential synergies by including a term for the two-way interaction between these variables in the regression models. All models were adjusted by baseline covariates. RII, SII and 95% confidence intervals were reported. The statistical analysis was performed using Stata v18.0 [28].

## Results

The final sample was constituted of 2,722 older adults. At baseline, 60.6% were female, the mean age was 64.9 (SD = 9.4), 9.4% were indigenous, and 55.0% had multimorbidity. Table 1 shows the distribution of our main exposures by IC trajectories. In comparison to individuals in class 1 (steep decreasing) and class 2 (moderate decreasing), older adults with a slightly increasing trajectory had higher levels of wealth (*p*-value < 0.01) and education (*p*-value < 0.01), and also displayed a lower proportion of women (*p*-value < 0.01). There were no significant differences in ethnicity.

**Table 1:** Distribution of wealth, education, gender, and ethnicity by intrinsic capacity trajectories

	Steep decreasing	Moderate decreasing	Slight increasing	*P*-value
	*n* = 463 (17%)	*n* = 1361 (50%)	*n* = 898 (33%)	
**Exposures**				
Wealth (permanent income quintiles)				
Q1 (poorest)	33.1	23.0	14.5	
Q2	20.8	20.6	13.1	
Q3	20.6	21.5	19.0	< 0.01
Q4	16.8	20.7	24.2	
Q5 (richest)	8.6	14.3	29.2	
Educational level (quintiles)				
Q1 (lowest)	49.0	25.6	9.9	
Q2	22.4	24.8	16.2	
Q3	20.8	32.1	31.2	< 0.01
Q4	4.5	8.9	17.6	
Q5 (highest)	3.4	8.6	25.2	
Gender (female = 1)	77.3	66.5	42.7	< 0.01
Ethnicity (indigenous = 1)	11.3	9.7	8.0	0.13

*P*-value for Chi-square test

Health and sociodemographic characteristics by IC trajectories are shown in Table 2. Older adults in class 3 (slight increase) were younger (*p*-value < 0.01), mostly with a couple (*p*-value < 0.01), paid job (*p*-value < 0.01), and health insurance (*p*-value < 0.01) than their counterparts in classes 1 and 2. They also displayed a significantly lower prevalence of multimorbidity (*p*-value < 0.01), higher levels of physical activity (*p*-value < 0.01), and lower sedentarism (*p*-value < 0.01).

**Table 2:** Sociodemographic and health characteristics by intrinsic capacity trajectories

	Steep decreasing	Moderate decreasing	Slight increasing	*P*-value
	*n* = 463 (17%)	*n* = 1361 (50%)	*n* = 898 (33%)	
**Sociodemographics**				
Age	73.6 (8.3)	65.5 (8.4)	59.6 (7.7)	0.02
Union status (with couple = 1)	47.6	63.4	76.2	< 0.01
Paid job	11.1	28.4	45.5	< 0.01
Health insurance	73.4	81.6	81.8	< 0.01
**Health and Lifestyle behaviors**				
Multimorbidity (≥ 2 chronic conditions)	64.4	59.2	45.2	< 0.01
*Physical activity*				
Low	53.8	44.4	36.2	
Moderate	23.0	25.6	26.7	< 0.01
High	23.2	30.0	37.1	
Sedentary behavior (daily sitting hours)	2.8 (2.9)	2.4 (2.1)	2.6 (2.1)	< 0.01
Sufficient fruits and vegetables intake	15.6	16.9	21.2	0.01
*Tobacco*				
Never	72.0	71.4	61.3	
Ever smoker, no longer	15.2	15.6	19.3	
Current smoker, not daily	5.7	3.9	6.1	< 0.01
Current smoker, daily	7.2	9.2	13.4	
*Alcohol consumption*				
Never	62.4	51.8	33.1	
Ever drinker, no longer	32.2	41.0	46.0	
Current drinker (low risk)	4.6	4.9	15.1	< 0.01
Current drinker (high risk)	0.9	2.2	5.9	

*P*-value for ANOVA or Chi-square tests

Table 3 shows the estimated inequalities in the IC trajectories for the three domains analyzed: SEP, gender, and ethnicity. Significant inequalities were observed in wealth and education. Individuals with the highest wealth and education level (quintile 5) were likelier to have moderately decreasing (wealth: SII = 0.09, CI95%:0.02–0.17; RII = 1.11, CI95%:1.02–1.19; education: SII = 0.18, CI95%:0.09–0.28; RII = 1.20, CI95%:1.09–1.32) or slightly increasing (wealth: SII = 0.13, CI95%:0.06–0.19; RII = 1.14, CI95%:1.07–1.21; education: SII = 0.31, CI95%:0.24–0.38; RII = 2.98, CI95%:2.47–3.60) trajectories than older adults with the lowest wealth and education (quintile 1), who were also more likely to be on the worst trajectory -steep decreasing.

**Table 3:** Inequality measures: absolute gap, relative gap, slope inequality index (SII), and relative inequality index (RII)

	**Moderate decreasing versus Steep decreasing**			**Slight increasing versus Steep decreasing**		
	Estimator	Confidence interval 95%		Estimator	Confidence interval 95%	
**Wealth**						
Absolute gap (Q5-Q1)	0.16	0.10	0.22	0.41	0.34	0.48
Relative gap (Q5/Q1)	1.24	1.14	1.35	1.91	1.67	2.18
Slope Inequality Index (SII)	0.09	0.02	0.17	0.13	0.06	0.19
Relative Inequality Index (RII)	1.11	1.02	1.19	1.14	1.07	1.21
**Educational level**						
Absolute gap (Q5-Q1)	0.27	0.21	0.34	0.65	0.59	0.71
Relative gap (Q5/Q1)	1.45	1.32	1.58	3.27	2.73	3.91
Slope Inequality Index (SII)	0.18	0.09	0.28	0.31	0.24	0.38
Relative Inequality Index (RII)	1.20	1.09	1.32	2.98	2.47	3.60
**Gender**						
Absolute gap (Female-Male)	-0.10	-0.14	-0.06	-0.32	-0.36	-0.27
Relative gap (Female/Male)	0.92	0.84	1.01	0.62	0.57	0.69
Slope Inequality Index (SII)	-0.17	-0.23	-0.11	-0.26	-0.31	-0.20
Relative Inequality Index (RII)	0.84	0.79	0.90	0.77	0.73	0.82
**Ethnicity**						
Absolute gap (Indigenous-non-Indigenous)	-0.03	-0.10	0.03	-0.09	-0.18	0.01
Relative gap (indigenous/non-indigenous)	0.95	0.87	1.05	0.87	0.74	1.01
Slope Inequality Index (SII)	-0.08	-0.15	-0.01	-0.06	-0.13	-0.01
Relative Inequality Index (RII)	0.92	0.86	0.99	0.94	0.89	0.99

RII and SII were estimated using generalized linear models with a logarithmic link function and an identity link function, respectively

There were also significant inequalities by gender and ethnicity. Men and non-indigenous people were more likely to have moderately decreasing (gender: SII = -0.17, CI95%: -0.23; -0.11; RII = 0.84, CI95%:0.79–0.90; ethnicity: SII = -0.08, CI95%: -0.15; -0.01; RII = 0.92, CI95%:0.86–0.99) or slightly increasing trajectories (gender: SII = -0.26, CI95%: -0.31; -0.20; RII = 0.77, CI95%:0.73–0.82; ethnicity: SII = -0.06, CI95%: -0.13; -0.01; RII = 0.94, CI95%:0.89–0.99) than women and indigenous people, implying that the latter were concentrated primarily in the steep declining trajectory.

**Fig. 1:**
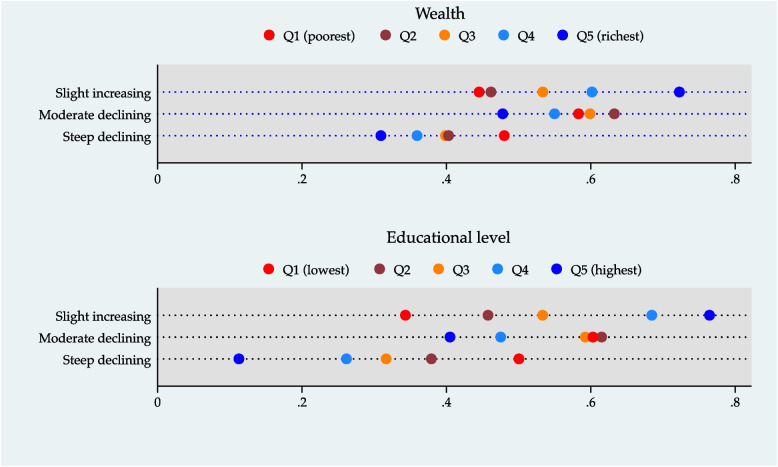
Socioeconomic inequalities in intrinsic capacity trajectories. Conditional probability for each intrinsic capacity trajectory given the wealth and educational level quintiles

**Fig. 2:**
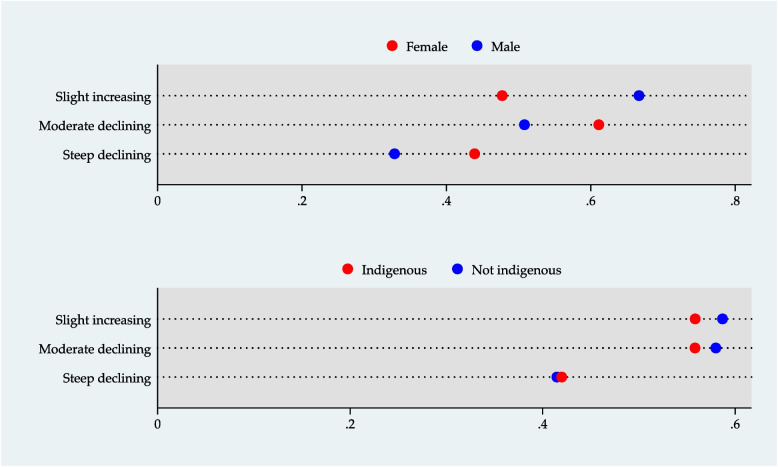
Gender and ethnicity inequalities in intrinsic capacity trajectories. Conditional probability for each intrinsic capacity trajectory given gender and ethnicity

Figures 1 and 2 depict the *equiplot* for wealth, educational level, gender, and ethnicity. A clear gradient is observed for all indicators according to the multinomial logistic regression results (Supplemental Table 2). The data shows that older adults with a lower level of wealth or education, who are women or indigenous people, have a greater likelihood of having an IC steeply decreasing trajectory concerning those with higher wealth, education, or who are men and non-indigenous. The results of the regression models evaluating interactions are shown in Supplemental Table 3. None of the interaction terms were significant.

## Discussion

Our findings corroborate the well-established evidence about the role of social factors in shaping socioeconomic health inequities. Based on a nationally representative sample of older Mexican adults, we observed that SEP, gender, and ethnicity were associated with different IC trajectories. In summary, we found that older adults with higher wealth and education, being men or non-indigenous, were more likely to have better trajectories than individuals with lower levels of wealth and education, being women or indigenous people. To the best of our knowledge, this study is the first attempt to examine the contribution of SEP, gender, and ethnicity to the socioeconomic health inequities associated even with the most inherent component of healthy aging: intrinsic capacity.

Our results suggest that SEP, using wealth and education as proxies, is a significant contributor to the process of healthy aging, reinforcing existing literature’s findings that these socioeconomic factors play crucial roles in determining health outcomes in later life and influencing the overall process of healthy aging [18, 29, 30]. However, previous studies have mainly evaluated the impact on functional capacity as a measure of successful aging. In contrast, we found that these disparities are even observable in the more comprehensive construct of intrinsic capacity.

In this study, we have used the novel concept of intrinsic capacity proposed by the WHO to reorient clinical practice and public policies associated with aging, and we have identified some degree of heterogeneity in their trajectories. The application of this concept represents several advantages for research and health policies in aging. First, intrinsic capacity is a comprehensive measure of health, which can be monitored throughout the life course and advocates for maintaining functional capacity in older adults, regardless of the presence of diseases [31]. Second, it focuses on functional capacity and its preservation rather than on the deficiencies of old age or the deterioration of bodily functions [32]. Third, it emphasizes identifying individual attributes associated with functionality and the influence of the environment in which people live [33]. These characteristics favor comparisons between countries and cultures about healthy aging and identifying contextual attributes that affect intrinsic capacity [34]. In this latter sense, customary monitoring of intrinsic capacity through longitudinal trajectories, for example, could help in early warning about a decline in functionality and inform potential preventive interventions.

The results of this study must be framed in the context of a middle-income country such as Mexico. Aging in Mexico has occurred amid a fragile economy marked by high levels of poverty and limited access to health services and resources [35]. This situation is further aggravated by the high prevalence of chronic conditions such as hypertension, diabetes, and hypercholesterolemia, but also by the presence of health conditions that mainly affect older adults, such as frailty, sarcopenia, and functional dependence, combined with conditions related to nutritional status like overweight/obesity and anemia [36]. Additionally, evidence shows that Mexican older adults from the most disadvantaged socioeconomic groups have worse health and nutritional conditions [37]. Despite these circumstances, our data show that 33% of older adults in Mexico maintained or even improved their intrinsic capacity over an 8-year observation period (2009 to 2017). This fact could be partially explained because, during the last 40 years, Mexico has implemented programs at the national level to alleviate poverty through conditional transfers [38], increase the coverage of health services [39], reduce food insecurity [40], and improve older people’s income through non-contributory pensions [41]. All in all, the results of our study show that significant inequalities (economic, gender, and ethnicity) associated with intrinsic capacity persist.

The beneficial effects of wealth and education can be attributed to several socio-biological mechanisms. Higher levels of education are generally linked to better health literacy and healthier behaviors during the entire life [42, 43], with variable impacts throughout the life course [44], which jointly affect health outcomes in late life. In addition, higher wealth and income (both related to education) may enable older adults to access better healthcare services and maintain healthier lifestyles [45]. In contrast to previous studies, our study notably underscores the role of socioeconomic factors in shaping the inherent potential for healthy aging. Our findings suggest that disparities, when manifested as differences in resources and opportunities, could profoundly affect this potential for aging healthily.

Gender and ethnic disparities in IC trajectories observed in our study further highlight the presence of health inequities in older adults. Women and indigenous individuals were found to be more likely to follow a steeply declining IC trajectory. This finding could be due to several factors, including differential access to resources, cultural barriers to healthcare, and systemic discrimination. Evidence has shown that women around the globe have a lower power position, less wealth and property, a higher burden of work of informal caring, less education, are employed in lower-paid jobs, and have less access to retirement benefits. Indigenous people have been systematically marginalized and isolated; they are poorer, with fewer years of education, less access to healthcare services, and higher unemployment rates [46–49]. These findings echo other studies that have reported gender and ethnic disparities in health-related outcomes. For instance, women lose more Disability Adjusted Life Years (DALYs) than men in reproductive infections, HIV, cancers, migraine, mental health, eye disorders, dementias, nutritional disorders, and muscle and bone conditions. Indigenous peoples have a lower life expectancy, higher infant mortality rates, infectious diseases like tuberculosis, diabetes, cancer, malnutrition, and a higher risk of mental illness like post-traumatic stress disorder and social phobia [50–53]. Our results underscore the need for targeted interventions to address these health inequities in early life and emphasize the need to identify the determinants of these gender and ethnic inequities, which can be modifiable by reducing gender and ethnic discrimination, that affect participation in the overall structure of opportunities and access to resources (education, healthcare services, etc.) throughout life [54].

Aside from the mechanisms just described, it has recently been suggested that health inequities are manifested in worse health through intermediate biological processes. Specifically, socioeconomic disadvantages associated with SEP, gender, and ethnicity may be related to chronic stress that impacts chronic conditions modulated by physiological wear and tear due to inflammatory responses, impaired immune function, and epigenetic acceleration of aging [55]. Furthermore, research suggests that SEP could be associated with allostatic load (a composite measure of overall physiological strain), a significant result since it has been suggested that allostatic load could represent the biological substrate of intrinsic capacity [56].

This study has some limitations that warrant consideration when interpreting findings. First, we could not examine the trajectories of intrinsic capacity before the onset of old age. Future studies should investigate its behavior throughout the lifespan, identifying the effects of inequities at different stages of life. Second, our analysis did not consider other social determinants, such as occupation, geographical location, and access to healthcare services. Third, we focused on gender and ethnicity because we know that they do not change over time, and wealth captures the accumulation by the time individuals reach older adulthood. However, this social determinant would likely change over time. Forth, exploring the impact of other individual and collective social determinants on intrinsic capacity, and identifying their mechanisms, is a pending task that likely requires more prolonged periods of observation, considering cohorts from youth and conducting more measurements over time.

## Conclusions

Although our objective was not to establish causality, differences in intrinsic capacity among social groups may partly be causal. Nevertheless, beyond identifying causal associations, evidence about differences in intrinsic capacity between social groups is already significant from a Public Health perspective, regardless of the causal mechanisms involved. In that vein, the findings of this study emphasize the need for policies and interventions addressing social determinants of health to promote healthy aging. Previous studies have highlighted effective interventions in reducing inequalities in aging, in particular, greater access to health services and pension programs, contributory and non-contributory. Even so, there is a knowledge gap to determine if interventions such as universal healthcare, formal education, and social and employment support effectively reduce health inequalities [57]. The health inequities identified in this study should also be considered in the planning and implementing of policies aimed at maintaining IC in older adults. Future research is needed to understand better the mechanisms through which these social determinants influence IC trajectories and to develop and test interventions to mitigate these disparities.

### Supplementary Information

**Supplementary Material 1.**

## Data Availability

Data can be procured through a formal request to the World Health Organization Multi-Country Studies Data Archive via its online platform (http://apps.who.int/healthinfo/systems/surveydata/index.php/catalog).
