# Supplementary material for: Intrinsic capacity trajectories and socioeconomic inequalities in health: the contributions of wealth, education, gender, and ethnicity
Source: Int J Equity Health. 2024 Mar 11;23:48. doi: 10.1186/s12939-024-02136-0 (PMC10926672; doi:10.1186/s12939-024-02136-0)
Supplement: Supplementary file 1 — Supplementary Material 1. [file 12939_2024_2136_MOESM1_ESM.docx]

**Intrinsic capacity trajectories and socioeconomic inequalities in health: the contributions of wealth, education, gender, and ethnicity**

**Supplemental Table 1. Durable goods, dwelling characteristics, and services included in the household wealth index**

| Item |
| --- |
| Durable good |
| Television |
| Security system |
| Car |
| Bicycle |
| Built-in kitchen sink |
| Washing machine |
| Dishwater |
| Refrigerator |
| Mobile/cellular phone |
| Computer |
| HiFi or music center (stereo system) |
| Livestock (cattle, goats, pigs, poultry) |
| Motor bike |
| Second home |
| Dwelling characteristics |
| Number of rooms (without counting the bathrooms/ toilets or hallways/passage ways) |
| Type of floor (hard floor -tile, cement, brick, wood- vs. earth floor) |
| Type of wall (cement, brock, stone, wood vs. mud, thatch, plastic sheeting) |
| Toilet facility vs. no facilities or bush or field |
| Access to services |
| Water |
| Sanitation |
| Electricity |
| Internet |

**Supplemental Table 2. Multinomial logistic regression results**

|  | **Moderate declining versus Steep declining** | | **Slight increasing versus Steep declining** | |
| --- | --- | --- | --- | --- |
|  | **Risk Relative Ratio** | **Confidence interval 95%** | **Risk Relative Ratio** | **Confidence interval 95%** |
| Wealth (reference: Q1) |  |  |  |  |
| Q2 | 1.24 | [0.82, 1.89] | 1.08 | [0.63, 1.84] |
| Q3 | 1.26 | [0.82, 1.95] | 1.58 | [0.93, 2.70] |
| Q4 | 1.62 | [1.00, 2.60] | 2.72 | [1.54, 4.82] |
| Q5 | 2.20 | [1.19, 4.08] | 5.04 | [2.50, 10.16] |
| Educational level(reference: Q1) |  |  |  |  |
| Q2 | 1.77 | [1.22, 2.58] | 2.51 | [1.53, 4.12] |
| Q3 | 2.10 | [1.43, 3.07] | 3.98 | [2.45, 6.47] |
| Q4 | 2.53 | [1.24, 5.14] | 8.87 | [4.03, 19.52] |
| Q5 | 10.15 | [3.28, 31.37] | 55.17 | [16.88, 180.29] |
| Gender (female=1) | 0.27 | [0.16, 0.43] | 0.06 | [0.04, 0.12] |
| Ethnicity (indigenous=1) | 0.52 | [0.32, 0.87] | 0.47 | [0.26, 0.86] |
| Physical activity (reference: low level) |  |  |  |  |
| Moderate Level | 1.13 | [0.77, 1.65] | 1.27 | [0.81, 2.01] |
| High Level | 0.81 | [0.56, 1.15] | 0.95 | [0.62, 1.46] |
| Smooking habit (reference: never) |  |  |  |  |
| Ever smoked, no longer | 0.66 | [0.43, 1.02] | 0.64 | [0.39, 1.06] |
| Current smoker, not daily | 0.37 | [0.18, 0.77] | 0.31 | [0.13, 0.72] |
| Current smoker, daily | 0.89 | [0.48, 1.66] | 1.03 | [0.51, 2.05] |
| Alcohol consumption (reference: never) |  |  |  |  |
| Ever drinker, no longer | 1.21 | [0.85, 1.71] | 1.38 | [0.91, 2.10] |
| Current drinker (low risk) | 0.68 | [0.33, 1.39] | 1.42 | [0.66, 3.08] |
| Current drinker (high risk) | 2.31 | [0.54, 9.85] | 3.41 | [0.73, 15.87] |
| Fruit & vegetable consumption | 0.96 | [0.63, 1.45] | 1.26 | [0.78, 2.03] |
| Age | 0.87 | [0.86, 0.89] | 0.79 | [0.77, 0.81] |
| Paid job (last week) | 1.74 | [1.13, 2.67] | 1.63 | [1.01, 2.62] |
| Union status (with partner=1) | 0.99 | [0.72, 1.36] | 1.21 | [0.82, 1.79] |
| Health insurance (yes=1) | 1.74 | [1.22, 2.49] | 1.97 | [1.27, 3.05] |
| Sedentary Behavior (Daily sitting hours) | 0.92 | [0.86, 0.99] | 0.92 | [0.85, 1.00] |
| Multimorbidity | 0.67 | [0.49, 0.93] | 0.42 | [0.29, 0.61] |

**Supplemental Table 3. Multinomial logistic regression results for the interaction terms**

|  | **Moderate decreasing versus Steep decreasing** | | **Slight increasing versus Steep decreasing** | |
| --- | --- | --- | --- | --- |
|  | Coefficient | p-value | Coefficient | p-value |
| **Main terms** |  |  |  |  |
| Wealth | -0.19 | 0.47 | 0.24 | 0.07 |
| Educational level | 0.08 | 0.76 | 0.26 | 0.05 |
| Gender | -0.19 | 0.08 | -0.27 | 0.00 |
| Ethnicity | -0.02 | 0.88 | 0.12 | 0.22 |
| **Interaction terms** |  |  |  |  |
| Wealth*Educational level | 0.18 | 0.49 | -0.11 | 0.41 |
| Wealth*Gender | 0.29 | 0.31 | 0.04 | 0.77 |
| Wealth*Ethnicity | -0.57 | 0.22 | -0.28 | 0.13 |
| Educational level*Gender | -0.04 | 0.90 | 0.25 | 0.06 |
| Educational level*Ethnicity | 0.96 | 0.07 | 0.30 | 0.11 |
| Gender*Ethnicity | -0.12 | 0.53 | -0.24 | 0.12 |

Adjusted for covariates shown in Table 2

**Supplemental Figure 1. Flow diagram for the analytical sample**

Baseline sample (Wave 1-2009)

N=2,404

Excluded from follow-up (Wave 2)

n=642

273 Dead

369 Lost to follow-up

Wave 2-2014

N= 3022

Refreshed sample=618

Excluded from follow-up (Wave 3)

n=872

368 Dead

504 Lost to follow-up

Wave 3-2017

N=2937

Refreshed sample=255

Total sample

N=3277

Excluded from analytical sample: missing values on some covariates

n=555

Analytical sample

N=2722

With 3 measurements=2014

With 2 measurements=708

**Supplemental S1. Operationalizing of intrinsic capacity**

We used the five domains proposed by Cesari et al to construct an intrinsic capacity (IC) score applying the Item Response Theory (IRT). We adjusted a graded response model (GRM) given that variables used for each domain of IC are ordered responses.

**Domains of IC**

*Cognition*

The SAGE study included five standard cognitive tests that encompass different dominions of cognitive function, such as: verbal learning and recall (immediate and delayed verbal recall), attention and working memory (forward and backward digit span), and executive function (verbal fluency). A brief description for each test procedure is given below.

1) Immediate and delayed verbal recall. The interviewers read a list of 10 words and asked the participants to immediately recall and repeat as many words as they could in one minute. Three trials of this assessment were performed. Upon completing the third trial, the interviewer administered the other cognitive tests, after which delayed recall ability was determined by asking subjects to remember the list of words.

2) Forward and backward digit span. In these tests, participants were required to repeat progressively longer series of numbers; the total score was recorded as the longest digit span repeated without error. The process was then performed with the older adult repeating a new set of increasingly longer digit spans in reverse.

3) Verbal fluency test. Consisted of naming as many animals (without using proper nouns) as possible in one minute; the final score was correct responses minus errors.

Composite z-scores were calculated (to facilitate the comparison of cognitive test performance between individuals) for each cognitive test and to calculate an overall composite cognitive function score. Specifically, z-scores for each cognitive test were first computed (using the sample mean) and these five z-scores were then summed to generate an overall composite cognition z-score. Finally, quintiles for the overall composite cognition z-score were created, with the 5th quintile reflecting the better cognition category.

*Psychological (mood)*

Depression was assessed using 18 questions derived from the World Mental Health Survey version of the Composite International Diagnostic Interview covering the presence of 10 depression symptoms within the prior 12 months. The International Classification of Diseases tenth revision (ICD-10) criteria of depression was used to determine respondents with major depressive disorder (depression). According to the ICD-10, assessment of depression uses two criteria: 1) Reported at least four of the 10 symptoms present for most of the day (almost every day) or lasting for more than two weeks, and 2) at least two of the following symptoms are present: depressed mood, loss of interest, and fatigability. In addition, respondents who reported taking any medication or other treatment for depression during the last 12 months were also categorized as having depression. Since we are generating the intrinsic capacity construct (which emphasizes physical and mental attributes, in a positive sense), this variable was coded as follows: 1 with no depression, and 0 with depression.

*Sensory*

Sensory impairments (poor vision and hearing capacity) have important implications for the health status and functioning of the individual. In this study we used the following four questions related to visual and hearing difficulties:

Vision

1) In the last 30 days, how much difficulty did you have in seeing and recognizing an object or a person you know across the road (from a distance of about 20 meters)?

2) In the last 30 days, how much difficulty did you have in seeing and recognizing an object at arm's length (for example, reading)?

Hearing

3) In the last 30 days, how much difficulty did you have in hearing someone speak across the room in a normal tone of voice (even wearing a hearing aid)?

4) In the past 30 days, how much difficulty did you have in hearing what was being said in a conversation with another person in a quiet room (even wearing a hearing aid)?

These four items were evaluated using a Likert-type scale with five response options: 1) None, 2) Mild, 3) Moderate, 4) Severe, 5) Extreme / Cannot do. Using the reverse coding, a global sensory capacity score was constructed (range 4-20), where higher scores imply greater capacity.

*Vitality*

We used two variables related to this domain: body mass index (BMI) and handgrip strength.

BMI was coded in five categories as follows: 1) Underweight: Below 18.5/Obesity class III: above 40; 2) Obesity class II: 35.0–39.9; 3) Obesity class I: 30.0–34.9; 4. Pre-obesity: 25.0–29.9; 5) Normal weight: 18.5–24.9. Because underweight and obesity class III are associated with low intrinsic capacity were collapsed in just one category.

Handgrip strength was measured twice for both hands with the use of the hand dynamometer (Baseline Electronic Smedley Hand Dynamometer, Fabrication Enterprises, White Plains, NY, USA). Quintiles for the grip strength (kg) were created, with the 5th quintile reflecting the higher strength.

*Locomotor*

Gait speed (met/sec), based on height, age, and sex-stratified values, was used as indicator of this domain. Four meters time walk was used to measure the gait speed. Participants were asked to walk at a normal pace. Walking aids (like cane) were allowed if the participant was more comfortable with it. Also, with this variable we generated its quintiles, where the 5th quintile expresses a greater locomotive capacity.

Graded response model

To capture the underlying latent construct of IC, we generated a measurement model. This measurement model includes parameters that represent the difficulty and the discriminatory power of each item associated with each domain of IC. In this approach, the items are allowed to differ in their relative difficulty and discrimination ability, which allows to create a common metric of IC. Specifically, we employed the Item Response Theory (IRT) and estimated a Graded Response Model (GRM). In the GRM, item responses are categorical and ordered, and is defined in terms of cumulative probabilities. The GRM estimation process proceeded as follows. We adjust an independent GRM for each round (with the same specification) and a score related to the latent trait (IC) was obtained. The final extracted IC score was transformed in a scale 0–100 with higher scores indicating better IC.

1. Cesari, Matteo et al. “Evidence for the Domains Supporting the Construct of Intrinsic Capacity.” The journals of gerontology. Series A, Biological sciences and medical sciences vol. 73,12 (2018): 1653-1660. doi:10.1093/gerona/gly011.
